# Ultra-Sensitive Gas Sensor Based on CDs@ZnO

**DOI:** 10.3390/s25030905

**Published:** 2025-02-02

**Authors:** Shuo Xiao, Zheng Jiao, Xuechun Yang

**Affiliations:** School of Environmental and Chemical Engineering, Shanghai University, Shanghai 200444, China; sxiao83@126.com (S.X.); xuechunyang@i.shu.edu.cn (X.Y.)

**Keywords:** CDs, ethylene glycol, sensor

## Abstract

Ethylene glycol (EG) is a colorless and odorless organic compound, which is an important industrial raw material but harmful to the environment and human health. Thus, it is necessary to develop high-performance sensing materials to monitor EG gas. Herein, sea urchin-shaped ZnO was successfully synthesized by a hydrothermal method. Subsequently, a series of carbon dot (CD)-modified ZnO nanocomposites were successfully prepared using a simple mechanical grinding method. The prepared CDs@ZnO-1 sensor exhibits an excellent response to EG gas, with a response value of 1356.89 to 100 ppm EG at the optimal operating temperature (220 °C). After five cycles of detection, the sensor can still maintain a stable response. The enhanced sensing performance of EG can be attributed to rich oxygen vacancies that are generated on the surface of CDs@ZnO, and the heterojunction formed between p-type CDs and n-type ZnO. This study provides inspiration for the development of high-response semiconductor metal oxide sensors.

## 1. Introduction

With the vigorous development of the industry, the emission of volatile organic compounds (VOCs) has attracted attention. Ethylene glycol (EG), as one of the VOCs, is usually used as a humectant [1], antifreeze, diluent [2], and textile auxiliary [3]. EG is a colorless, odorless, sweet-tasting liquid, but it can be deadly to the human body [4]. EG vapor has a certain toxicity that can cause dizziness, headaches, nausea, abdominal pain, dry mouth, dry tongue, and cold sweat if inhaled. It can harm the kidney, liver, stomach, intestine, and other internal organs of humans and animals, impair reproductive function, metabolism, and the immune system, and cause hemoglobin concentration to decline [5]. As a result, it is critical to develop a gas sensor capable of detecting EG quickly and effectively.

Currently, researchers have developed a variety of techniques for detecting EG gas, such as gas chromatography-mass spectrometry (GC-MS) [6], capillary column gas chromatography (CCGC) [7], gas chromatography (GC) [8,9], and so on. However, the size of these instruments has a lot of drawbacks. Gas sensors stand out due to their low cost, miniaturization, integration, and real-time detection.

Gas sensing materials are the most significant component of gas sensors, and Metal Oxide Semiconductor (MOS) sensing materials have received the most attention [10,11]. For example, Su et al. developed a feasible hydrothermal technique for the synthesis of a hierarchical dendritic CuO/Co_3_O_4_ nanowire heterostructure, which exhibits a strong affinity for EG adsorption. The response of a gas sensor based on the hierarchical CuO/Co_3_O_4_ heterostructure to 100 ppm EG is as high as 6.3 at 130 °C. The enhanced sensing performance toward EG can be attributed to the formation of a unique CuO/Co_3_O_4_ core–shell heterojunction structure [12]. MOSs are mainly divided into n-types and p-types. ZnO is the most widely used n-type MOS material. It is regarded as an excellent gas sensing material due to its wide band gap, non-toxicity, good thermal and chemical stability, high electron mobility, and response range to oxidizing and reducing gases [13,14,15,16]. For example, Ding et al. prepared ZnO/rGO nanosheets by combining chemical precipitation with a hydrothermal method. At the optimal operating temperature of 220 °C, the response of the gas sensor to 100 ppm EG is as high as 277. Compared with an intrinsic ZnO gas sensor, the working temperature is reduced and the response is increased by 1.1 times. The ZnO/rGO sensor has a fast response/recovery time of approximately 38 s/26 s and exhibits excellent cyclic repeatability and stability. Its detection limit is as low as 1 ppm. The excellent gas sensing properties are mainly attributed to the large specific surface area and more active sites in ZnO/rGO nanosheets [17]. Moreover, the MOF template method is commonly used to prepare ZnO with diverse and adjustable morphologies. For example, Kou et al. prepared the In_2_O_3_@ZnO-based gas sensor using MIL-68 (In)@ZIF-8 as the sacrificial template to test its sensing capability for EG. The In_2_O_3_@ZnO-based sensor has an ultra-high response (20 ppm-200.12) and excellent selectivity towards EG. The heterojunction designed and constructed with dual MOFs is the main reason for improving the EG response of sensors [18].

In this work, sea urchin-shaped ZnO was successfully synthesized. Subsequently, carbon dots (CDs) were selected as ZnO-modified nanomaterials due to their flexible surface states [19,20]. A series of CD-modified ZnO nanocomposites were successfully prepared using a simple mechanical grinding method. The gas sensitive materials were studied through morphology and structural characterization systems, and their detection performance for EG was systematically tested. Finally, the mechanism by which CD modification effectively improves the gas sensing performance of ZnO-based sensors has been revealed.

## 2. Materials and Methods

### 2.1. Materials

Zinc chloride (ZnCl_2_), polyvinylpyrrolidone (PVP), sodium hydroxide (NaOH), urea, and *p*-phenylenediamine were purchased from Sinopharm Chemical Reagent. All chemicals used in this study were of analytical grade and without any further purification.

### 2.2. Preparation of ZnO

First, 0.05 mol of ZnCl_2_ and 0.0001 mol of PVP were dissolved in 100 mL of deionized water. After 30 min of magnetic stirring, 0.05 mol of NaOH was added to the above clarified solution and vigorously stirred for 1 h. The solution was then transferred to a 100 mL Teflon-lined stainless steel reactor and kept in an oven at 180 °C for 8 h. The reaction solution was repeatedly cleaned and centrifuged with ethanol and deionized water after it had cooled to room temperature. After that, it was dried for 12 h in a vacuum oven at 60 °C. The dried powder was put into a mortar for grinding, and then put into a muffle furnace, heated to 500 °C at a rate of 2 °C/min in the air for 2 h. The white powder obtained after natural cooling is the prepared ZnO.

### 2.3. Preparation of CDs@ZnO

In a typical procedure, 3.0 g of *p*-phenylenediamine was combined with a certain quantity of urea and dissolved in 100 mL of deionized water. The mixture was then transferred to a Teflon-sealed autoclave and heated at 180 °C for 3 h. After cooling to room temperature, the obtained rough CD solution was centrifuged and the supernatant was kept for dialysis. After freeze drying, CDs’ powder was obtained.

In order to maximize the retention of the material properties of CDs, CDs@ZnOs were prepared by co-mixing mechanical grinding. First, 0.1 mg of CDs were dissolved in 10 mL water to obtain the yellow solution. The 100 μL CD solution was defined as 1 equivalent (Equation (1)). Equations (1) and (2) of CD solution were dropped into 1 mg ZnO using a pipette and fully stirred for 30 min to prepare CDs@ZnO-1 and CDs@ZnO-2, respectively. The sample without CDs is pure ZnO.

### 2.4. Characterization

The crystal structure of samples was characterized using an X-ray powder diffractometer (XRD) (D/max-2200, Rigaku, Tokyo, Japan). Scanning electron microscopy (SEM) (Hitachi Regulus 8100, Tokyo, Japan) was used to characterize the samples’ morphology. X-ray photoelectron spectroscopy (XPS, Kalpha, Thermo, MA, USA) was utilized to characterize the chemical composition of samples. The N_2_ adsorption-desorption isotherm was determined using an automated surface analyzer (Quadrasord SI, Quantachrome, FL, USA). The specific surface area of the sample powder was determined by the Brunauer, Emmett, and Teller (BET) methods, and the porosity was determined by the Barrett, Joyner, and Holenda (BJH) methods. The experiment considered two steps: processing the sample to remove moisture and impurities (step 1), and analyzing the surface area by the BET method (step 2). The samples’ Raman spectra were examined using Raman microscopy (Invia, Renishaw, London, UK).

### 2.5. Fabrication and Measurements of Gas Sensors

(1) Coating: 1 mg powder sample was placed in the mortar, along with an appropriate amount of turpentine alcohol and ethanol, and ground for 1 h to form a uniform paste. The paste was then uniformly coated with a scraper on the surface of a hollow Al_2_O_3_ ceramic tube to form a thick film of appropriate thickness and dried in an oven.
(2) Welding: The four Pt wires on the surface of the Al_2_O_3_ ceramic tube are unfolded, and the two pairs of Pt wires on the gold electrode of the ceramic tube are welded to the sensor base with an electric iron. After that, a Ni-Cr alloy heating wire is inserted in the middle of the ceramic tube and also welded to the sensor base to form a complete component.
(3) Aging: After the gas sensitive sensor is made, it is inserted into the test circuit board and placed on the aging table at 100 °C for continuous aging for 2 days to improve the stability of gas sensing. The WS-30A test system was used to fully test the gas sensitivity of the material.

### 2.6. Gas Sensing Experimental Setup

All gas sensing tests are conducted using the WS-30A gas sensor test system made by Weisheng Electronic Technology Co., Ltd., Zhengzhou, China. The test system is mainly composed of test box and test data analysis software. The test box includes an 18 L air distribution box, mixing fan, evaporator, test voltage adjustment knob, heating voltage adjustment knob, test circuit board, resistance load card, etc. The test system mainly reflects the characteristics of the gas sensor by measuring the voltage on the load resistance in series with the gas sensor to obtain the resistance and other parameters of the gas sensing material. The adjustment of the heating voltage can change the working temperature of the gas sensor. The test method of the gas sensor is a static test method. The gas distribution formula is as follows:

(1) $$V_{x}=\frac{V\;\times \;C\;\times \;M}{22.4\;\times \;d\;\times \;p}\;\times \;10^{-9}\;\times \;\frac{273\;+\;T_{R}}{273\;+\;T_{b}}$$

In the formula, V represents the volume of the test box (mL), and the volume of the gas distribution box used in this experiment is 18 L (300 mm × 300 mm × 200 mm); C represents liquid vapor concentration (ppm); M represents liquid molecular weight (g); d represents liquid specific gravity (g/cm^3^); p represents liquid purity; T_R_ represents room temperature (°C); and T_b_ represents the temperature in the test chamber (°C).

The response value of the sensor was defined as the ratio of Ra/Rg, where Ra and Rg refer to the resistance values of the sensor in the air and target gas, respectively.

## 3. Results

### 3.1. Characterization Results

**Figure 1:**
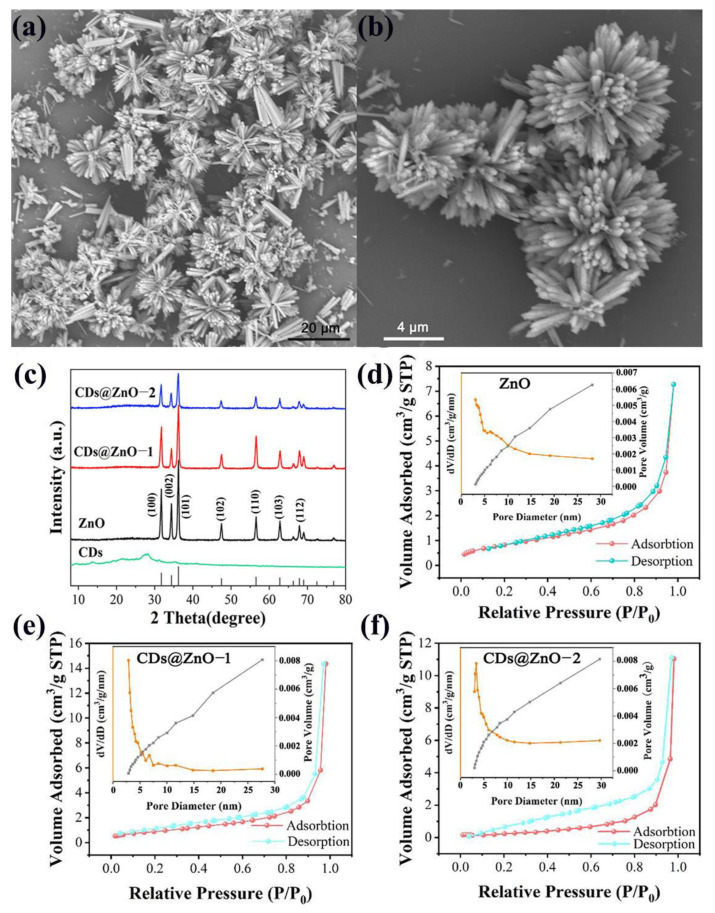
The SEM images of (**a**) ZnO and (**b**) CDs@ZnO-1. (**c**) The XRD spectra of CDs, ZnO, and CDs@ZnO. The nitrogen adsorption–desorption isotherm and BJH pore size distribution of (**d**) ZnO, (**e**) CDs@ZnO-1, and (**f**) CDs@ZnO-2.

The ZnO and CDs@ZnO were successfully prepared and their morphology was evaluated using clear and high-quality SEM and TEM images. Figure 1a shows the SEM image of ZnO, which is sea urchin-shaped and composed of micro columns approximately 3 µm in length. As shown in Figure 1b, the morphology of CDs@ZnO-1 did not show significant changes compared to pure ZnO, indicating that CDs only occur on the surface of ZnO and do not enter the ZnO lattice, causing changes in the morphology of ZnO. In Figure S2a, it can be seen that the CDs are compounded on ZnO, and the measured crystal plane spacing is 0.241 nm, corresponding to the (101) crystal plane of hexagonal fibrous ZnO [14]. Figure 1c exhibits the XRD patterns of ZnO, CDs@ZnO, and CDs, respectively. All characteristic diffraction peaks of the ZnO and CDs@ZnO samples are matched well with the XRD patterns of wurtzite ZnO (PDF#89-1397) with the hexagonal crystal structure, the corresponding lattice constants are a = b = 3.243 Å, c = 5.195 Å, and the space group is P63mc [21]. The diffraction peaks at 2*θ* = 31.73°, 34.38°, 36.21°, 47.48°, 63.77°, and 67.86° correspond to the (100), (002), (101), (102), (110), (103), and (112) crystal planes of ZnO, respectively [22]. No peaks caused by other impurities were detected, indicating that the purity of the samples is high. The sharp diffraction peaks of CDs at 2*θ* = 20–30° are detected, and the wide diffraction peaks of CDs@ZnO-1 and CDs@ZnO-2 at 2*θ* = 20–30° also can be observed. The wide diffraction peaks grow more apparent as the amount of CD doping rises (Figure S1). The pore structure and specific surface area of the ZnO and CDs@ZnO samples are analyzed. Figure 1d–f show the nitrogen adsorption and desorption curves for ZnO, CDs@ZnO-1, and CDs@ZnO-2, respectively. According to the IUPAC classification, all three samples belong to the typical type IV isotherm. The specific surface areas of ZnO, CDs@ZnO-1, and CDs@ZnO-2 are 2.575 m^2^/g, 3.268 m^2^/g, and 3.969 m^2^/g, respectively. As shown in the inset of Figure 1d, the pore size of pure ZnO is mainly concentrated around 5.5 nm. The aperture of CDs@ZnO-1 has slightly increased, with a strong peak around 7 nm (as shown in Figure 1e). However, as the composite ratio of CDs continues to increase, CDs may agglomerate and block the pores; therefore, the pore size of CDs@ZnO-2 has significantly decreased, and is mainly concentrated at 3.5 nm, as shown in the inset of Figure 1f. All three samples are mesoporous materials, and the composite of CD and ZnO has little effect on the morphology of samples.

**Figure 2:**
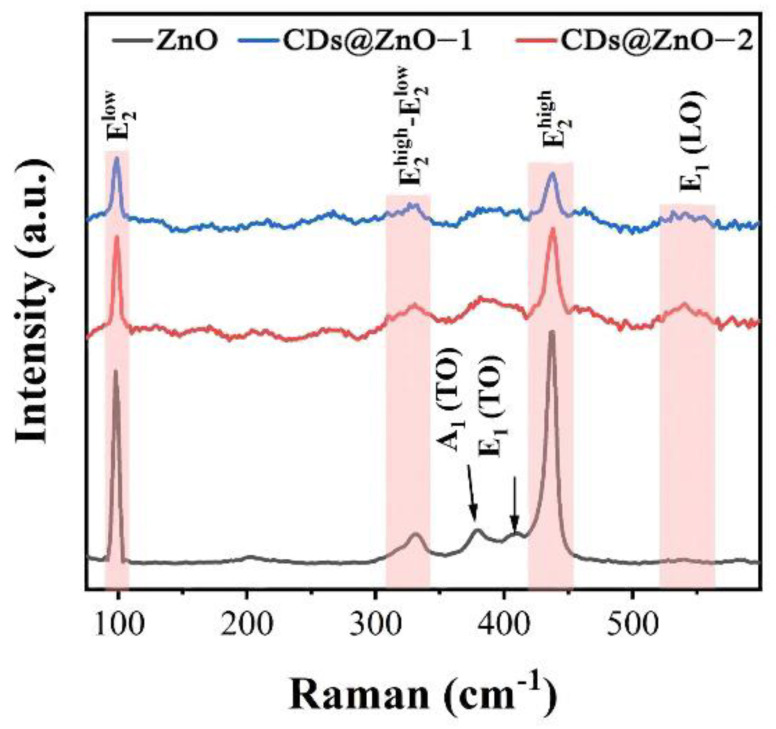
Raman spectra of ZnO, CDs@ZnO-1, and CDs@ZnO-2.

Figure 2 shows the Raman spectra of ZnO, CDs@ZnO-1, and CDs@ZnO-2. The three detectable peaks at 330 cm^−1^, 379 cm^−1^, and 436 cm^−1^ are attributable to ZnO, which can be assigned to the $E_{2}^{\mathrm{high}}-E_{2}^{\mathrm{low}}$ multiphonon processes, A_1_(TO) and $E_{2}^{\mathrm{high}}$, respectively [23]. In addition, the peak at 566 cm^−1^ corresponds to the E_1_(LO) mode of ZnO due to structural defects in the ZnO lattice [24]. Two high-intensity Raman peaks at 99 cm^−1^ and 437 cm^−1^ are attributed to the $E_{2}^{\mathrm{low}}$ vibration mode of the Zn sublattice vibrations and to the $E_{2}^{\mathrm{high}}$ vibration mode attributed to the oxygen atom, respectively [25]. These two modes are nonpolar because there is no net-induced polarization due to the displacement of the ions. The Raman peak at 380 cm^−1^ is associated with the A_1_(TO) polar mode. Raman analysis clearly demonstrates the formation of Zn-O in the crystal and its activity mode.

**Figure 3:**
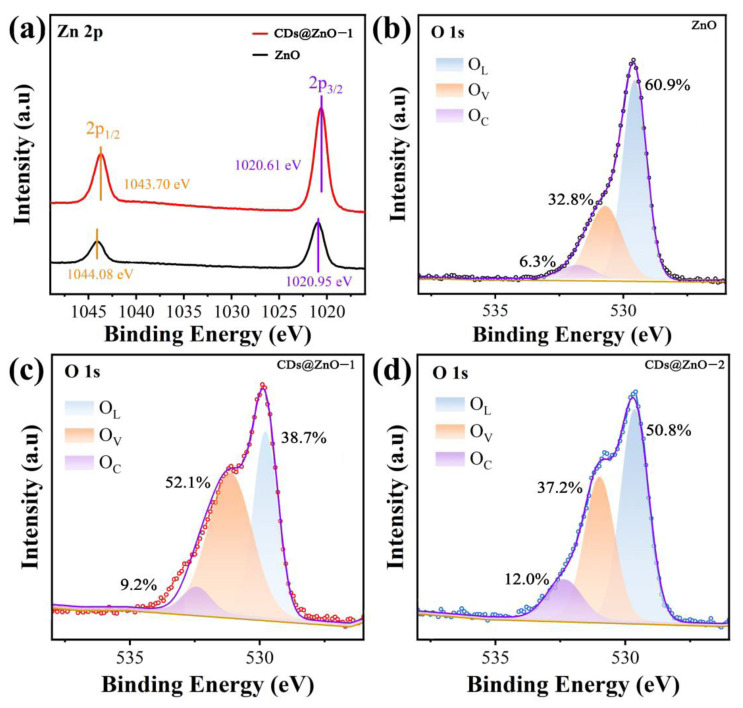
(**a**) The XPS Zn 2p spectra of ZnO and CDs@ZnO-1. The XPS O 1s spectra of (**b**) ZnO, (**c**) CDs@ZnO-1, and (**d**) CDs@ZnO-2, the circles represent the raw data, and the lines represent the fitting result.

The defect states of the materials were characterized by XPS. As shown in Figure S3a, ZnO mainly contains Zn and O elements. After composite CDs, the N element was added to CDs@ZnO. It is because CDs contain a small amount of the N element. The C1s spectra show a significant increase in oxygen-containing functional groups on the surface of CDs@ZnO-1 compared to ZnO [17]. Figure 3a shows two symmetric peaks in the Zn 2p XPS fine spectra of both ZnO and CDs@ZnO-1, proving that the sample’s zinc ion is a single type of Zn^2+^ [26]. The peaks centered at 1020.95 eV and 1020.61 eV correspond to Zn 2p_3/2_, and the peaks centered at 1044.08 eV and 1043.70 eV correspond to Zn 2p_1/2_ [27]. The distance between the two peaks is about 23.0 eV, which is consistent with the reported data [28]. Additionally, the binding energy of Zn in CDs@ZnO-1 is moved to the lower direction, indicating the presence of electronic interactions between ZnO and CDs [29]. When there is electron transfer between CDs and ZnO, more abundant electrons can be generated on the surface of CDs@ZnO. High concentrations of electrons often result in high gas sensing response signals and low optimal operating temperatures for sensors [30]. The Mott–Schottky (M-S) curve demonstrates the successful formation of a heterojunction between CD and ZnO (Figure S2b). The inverted “V”-shaped M-S curve is a characteristic of forming heterojunctions. The M-S curve of CDs@ZnO-1 shows a clear inverted “V” shape, which strongly proves that appropriately loaded CDs can successfully construct p-n nanoheterojunctions at the material interface. The formation of p-n nanoheterojunctions can provide many effective channels for carrier transport, which is beneficial for the effective separation of electrons and holes [31]. Figure 3b–d show the O 1s fine spectra of the three materials. After fitting, the O 1s spectra can be divided into three peaks (O_L_, O_V_, O_C_). The peak at 529.7 eV is the lattice oxygen (O_L_), which is generally not reactive and stable in nature, and not an important indicator for judging gas sensing. The peak at 532.3 eV is the chemisorbed oxygen (O_C_), which is related to the reaction temperature. Meanwhile, the peak at 531.1 eV is oxygen vacancy (O_V_), which can provide active sites for gas adsorption and reaction on the surface of sensing materials, and is decisive for the gas sensing characteristics of the materials [30]. The O_V_ contents of ZnO, CDs@ZnO-1, and CDs@ZnO-2 were 32.8%, 52.1%, and 37.2%, respectively. After the CD composite, the O_V_ content of CDs@ZnO-1 increased by 19.3% compared to pure ZnO. The reason is that local surface electron reconstruction occurs at the interface between CDs and ZnO, forming a heterojunction and promoting the generation of O_V_ [17,24]. However, as the composite ratio of CDs further increases, some CDs will agglomerate and become inactive on the surface of ZnO, resulting in a decrease in O_V_ content [32]. It can be seen that the oxygen defects increased after the introduction of CDs, and the CDs@ZnO-1 sample contained the most O_V_, which is beneficial to improve the gas sensing performance.

### 3.2. Gas Sensitive Properties

**Figure 4:**
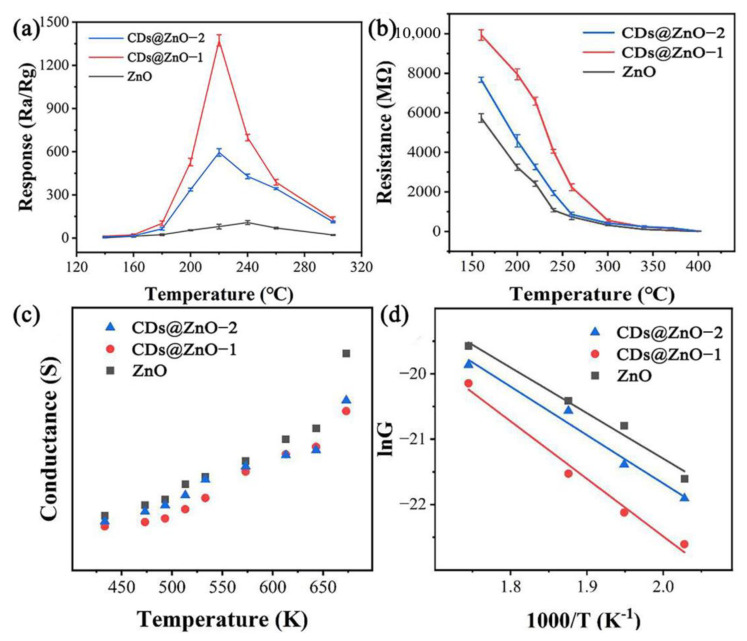
(**a**) The gas response of the ZnO and CDs@ZnO sensors towards 100 ppm EG at different operating temperatures. (**b**) Baseline resistance at different temperatures, (**c**) conductance versus temperature plot, (**d**) lnG-(1/T) plot and corresponding linear fit of ZnO- and CDs@ZnO-based sensors.

In practical applications, the performance of the gas sensor is affected by many factors, such as temperature and humidity. Figure 4a illustrates the correlation between the operating temperature and the gas sensor’s sensitivity. Three sensors respond to EG gas in the range of 140 °C to 300 °C. The sensitivity increases with temperature in the range of 140–220 °C. The CDs@ZnO-based sensors have the highest sensitivity at 220 °C, while ZnO-based sensors have the highest sensitivity at 240 °C. Subsequently, with the continuous increase in temperature, the sensitivity of the three sensors began to decline. The response of the three sensors shows a pyramid shape with the change in temperature. The reason is that at lower operating temperatures, the reaction between chemisorbed oxides and gas molecules cannot fully occur, lacking activation energy. On the contrary, excessively high temperatures can weaken the dynamic adsorption of target gases on the surface of sensitive materials, leading to a decrease in sensitivity [18]. The optimal working temperature (OOT) is close to the gasification temperature of EG (197.85 °C). Due to heat loss, the operating temperature of gas sensors is generally slightly higher than the gasification temperature of VOCs gas. Compared with ZnO-based sensors, the optimal operating temperature of CDs@ZnO-based sensors decreases, which proves that the addition of CDs not only greatly improves the sensitivity, but also reduces the optimal operating temperature. From the perspective of actual EG detection, the response of the CDs@ZnO-based sensor is high enough even if it works below 200 °C.

Table 1 compares the detection performance of the EG gas sensor. It can be found that CDs@ZnO-1-based sensors have obvious advantages in response recovery time and sensitivity, which will be of great value in practical applications.

Figure 4b shows the baseline resistance of ZnO, CDs@ZnO-1, and CDs@ZnO-2 sensors in air as a function of the operating temperature. The baseline resistance of all three sensors decreases with the increase in operating temperature. It is because that as the temperature increases, the binding of the nucleus to electrons decreases, more free electrons are produced, the carrier concentration increases, and the resistance decreases. The order of baseline resistance is R_CDs@ZnO-1_ > R_CDs@ZnO-2_ > R_ZnO_. With excessive CD loading, the CD nanoparticles on the ZnO surface will agglomerate to form independent electron transport channels. Due to the high conductivity of CDs, the initial resistance of CDs@ZnO-2 will be lower than that of CDs@ZnO-1. Figure 4c shows the relationship between conductivity and temperature. At the absolute temperature (*T*, *K*), the conductivity (*G*, *S*) of semiconductor oxide can be expressed by the Arrhenius formula in Equation (2) [31].

*G* = *G*_0_ exp(−*e V_S_*/*kT*) (2)

ln*G* = (−*e V_S_*/*k*) (1/*T*) + ln*G*_0_ (3)

*G_0_* is the finger-front factor of the interparticle conductivity, *eV_S_* is the thermal activation energy, and *k* is the Boltzmann constant (1.38 × 10^−23^ J/K). In a certain temperature range, *G_0_* and *eV_S_* can be regarded as temperature-independent constants, so Equation (2) can be rewritten as Equation (3). Figure 4d shows a linear correlation between the logarithm of conductivity and the reciprocal of temperature within the temperature range of 1.75 K^−1^ to 2.05 K^−1^. The fitted data are shown in Table 2. The activation energy of the CDs@ZnO-1 and CDs@ZnO-2 samples is 0.75 eV and 0.64 eV, higher than that of ZnO (0.60 eV). For n-type semiconductor gas sensitive materials, typically the higher their baseline resistance, the more significant the decrease in resistance, and the greater the device response intensity in reducing gases [33].

**Figure 5:**
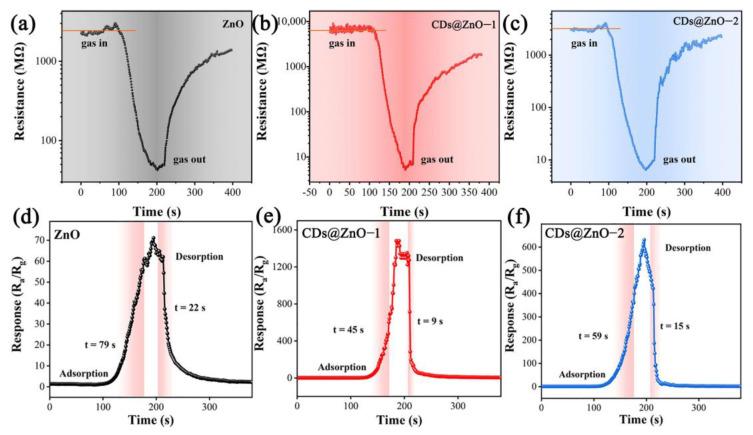
Real-time resistance curves of (**a**) ZnO sensor, (**b**) CDs@ZnO-1 sensor, and (**c**) CDs@ZnO-2 sensor. Response and recovery time of (**d**) ZnO sensor, (**e**) CDs@ZnO-1 sensor, and (**f**) CDs@ZnO-2 sensor.

Figure 5 shows the transient sensing curves of the ZnO and CDs@ZnO sensors for 100 ppm EG gas at the optimum operating temperature. As shown in Figure 5a–c, the resistance of all sensors drops rapidly in the EG gas, then gradually converges to a stable value, and returns to the initial resistance in the air after leaving the EG gas. The law of resistance variation is consistent with the characteristics of n-type semiconductors [34]. The three sensors have similar transient response recovery processes, indicating that all three samples can adsorb and desorb the gas effectively. The response recovery time can reflect the detection efficiency of the sensors. The response recovery time curves of the ZnO and CDs@ZnO-based sensors were analyzed as shown in Figure 5d–f. After injecting 100 ppm EG gas into the test chamber, the three sensors responded rapidly, and the response values decreased rapidly after removing the EG gas. The response/recovery times (t_a_/t_d_) of the three sensors were calculated to be 79 s/22 s, 45 s/9 s, and 59 s/15 s. The response and recovery times of the CDs@ZnO-1 sensor were the shortest, demonstrating that the composite of CDs may significantly minimize the response recovery time. In addition, it can be seen that the CDs@ZnO-1 sensor not only had a short response recovery time, but also had the highest response signal. The Ra/Rg values of the ZnO, CDs@ZnO-1, and CDs@ZnO-2 sensors were 67.11, 1356.89, and 638.10, respectively, indicating that the composite CDs can improve the sensitivity of the CDs@ZnO-based sensor by a maximum of 20 times.

**Figure 6:**
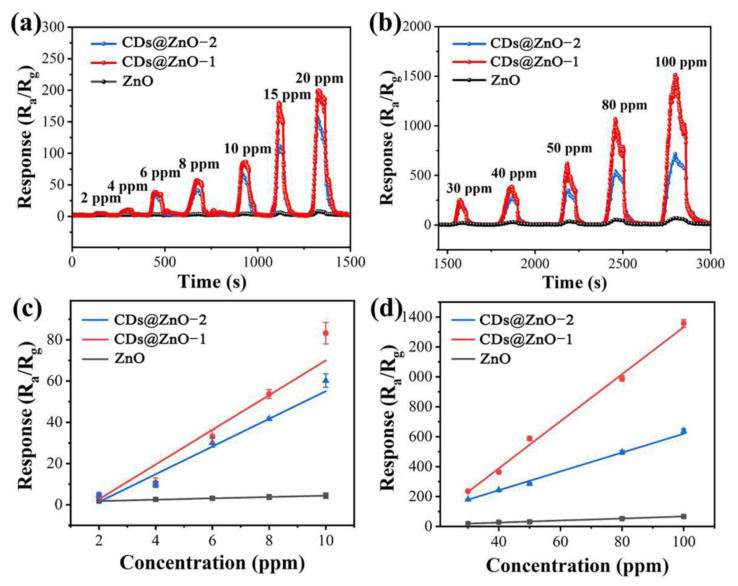
Plots of response gradients of ZnO and CDs@ZnO-based sensors for different concentrations of EG gas: (**a**) low concentration, (**b**) high concentration; fitted response curves of ZnO- and CDs@ZnO-based sensors for different concentrations of EG gas: (**c**) low concentration, (**d**) high concentration.

What is more, the transient response curves of all the samples under different concentration ranges (1–100 ppm) of EG gas were explored, and the results are shown in Figure 6. It can be seen that the responses of all samples increase step by step with the increase in the concentration of EG gas. Figure 6a,c and Table S1 show the gradient response curves and linear fitting results of three sensors at low concentrations (1–20 ppm). Figure 6b,d and Table S1 show the gradient response curves and linear fitting results of three sensors at high concentrations (30–100 ppm). At low and high concentrations, all three sensors have a good linear response, which is very convenient for stable testing in practical applications, the prediction of an unknown concentration of EG gas, and the estimation of detection limits.

**Figure 7:**
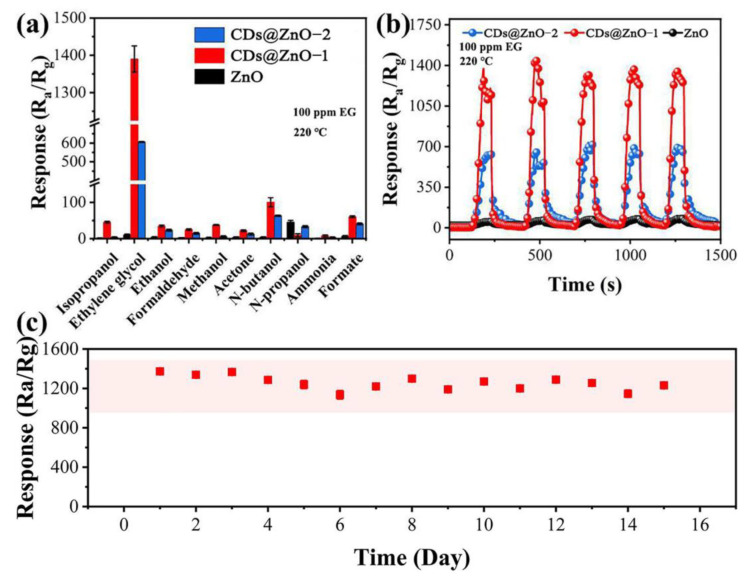
(**a**) Selectivity of ZnO and CDs@ZnO sensors for different gases at 100 ppm, (**b**) 5 cycles’ response of three sensors to 100 ppm EG gas at 220 °C, (**c**) stability test of CDs@ZnO-1-based sensor over a week.

Selectivity is an important characteristic parameter for gas sensitive sensors, and whether the sensor needs to respond to only one gas or to multiple gases in practical applications is also an important idea in the design of sensing materials. In Figure 7a, under the same test conditions, the CDs@ZnO-based sensor prepared in our work responded significantly better to EG than to the other nine VOC test gases, indicating its outstanding selectivity. The reason may be that the dissociation bond energy of each gas is different, as follows: EG (354.8 kJ/mol) < N-propanol (400.1 kJ/mol) < acetone (401.2 kJ/mol) < ammonia (427.0 kJ/mol) < methanol (440.2 kJ/mol) < ethanol (441.0 kJ/mol) < formate (493.0 kJ/mol) < formaldehyde (523.7 kJ/mol) < N-butanol (551.2 kJ/mol) [35,36]. EG has the lowest bond dissociation energy and is more prone to reactions. Additionally, due to the presence of two hydroxyl groups in EG, oxidation reactions are also more likely to occur. Figure 7b shows the response recovery curves of the three sensors to the same concentration of EG gas. After five consecutive cycles, the resistance of all sensors can still quickly respond and recover, and the response values remain stable. The stability of the CDs@ZnO-1 sensor in 15 days is shown in Figure 7c. The response value fluctuates within a certain error range, indicating that the sensor has ideal repeatability and long-term stability.

**Figure 8:**
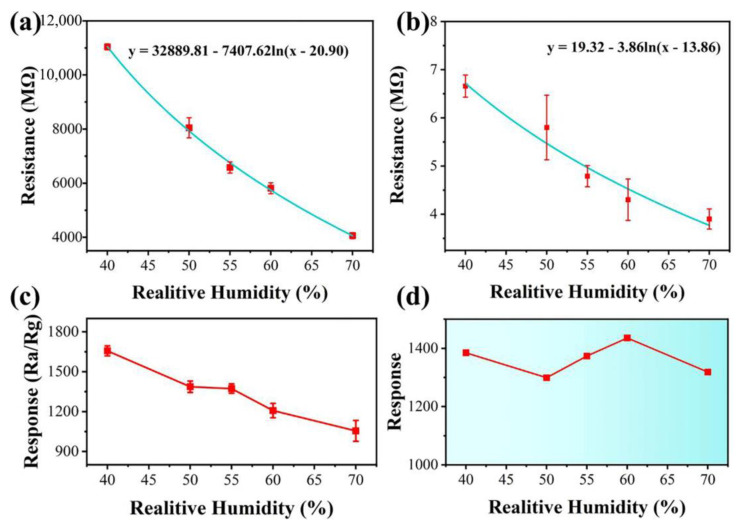
The properties of CDs@ZnO-1: (**a**) the fitted resistance curve in the air and (**b**) in 100 ppm EG under different humidity. (**c**) Responses of sensor to 100 ppm EG under different humidity. (**d**) Responses under different humidity after compensation.

Figure 8a,b show that the resistance changes of the CDs@ZnO-1 sensor are related to the humidity in air and EG gas, respectively. The resistance decreases and the conductivity increases as the humidity increases. Figure 8c shows that the response of the CDs@ZnO-1 sensor decreases with increasing humidity. For practical applications, humidity compensation can be introduced to eliminate significant differences in response at different humidity levels. The resistance can be fitted with the logarithmic function, using a value of RH below 55% as the compensation criterion. Resistance compensation can be expressed as (4) and (5) [30]:

R_a-Actual_ = R_a-Record_ + a_1_ln((H − b_1_)/(50 − b_1_)) (4)

R_g-Actual_ = R_g-Record_ + a_2_ln((H − b_2_)/(50 − b_2_)) (5)

where H is the relative humidity, and a_1_, b_1_, a_2_, and b_2_ represent the coefficients of the fitted curve, which are 7407.62, 20.90, 3.86, and 13.86, respectively. R_a-Record_ and R_g-Record_ represent the resistance recorded in the air and EG gas, respectively. R_a-Actual_ and R_g-Actual_ represent the compensated resistance in the air and EG gas, respectively. Finally, the calibration resistance will be calculated by Equation (6).

R_actual_ = R_g-Actual_/R_a-Actual_ (6)

The calibrated resistance value was 24.97, 28.26, 20.54, and 24.34 at 40%, 50%, 55%, 60%, and 70% relative humidity, respectively, as shown in Figure 8d. Resistance calibrated for humidity has more advantages in practical applications.

### 3.3. Gas Sensitive Mechanism

**Figure 9:**
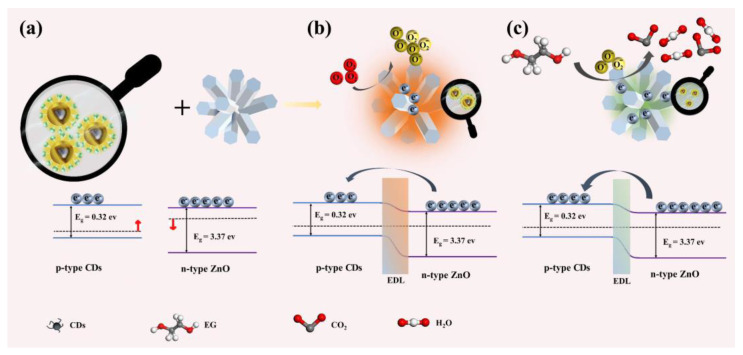
The schematic diagram for gas sensing mechanism of the CDs@ZnO-based sensor, (**a**) schematic diagram of band structure of CDs and ZnO, the electron transfer process at the surface interface of CDs@ZnO (**b**) in air and (**c**) EG gas.

It is commonly known that the Fermi energy level of the n-type semiconductor ZnO is close to the valence band, while the Fermi energy level of the p-type semiconductor CD is close to the valence band [35]. The electrons “diffuse” from the high Fermi energy level to the low Fermi energy level, and the Fermi energy level position changes to reach an equilibrium state, when CDs are combined with ZnO. Therefore, the band bending of both forms a p-n heterojunction. The CDs@ZnO is an n-type semiconductor because ZnO is the host material, and its electron concentration determines the resistance of the semiconductor (Figure 9a and Figure S2b) [33].

When CDs@ZnO is exposed to high temperatures, oxygen molecules from the surrounding air can be adsorbed on its surface to produce adsorbed oxygen molecules. These adsorbed oxygen molecules are then transformed into reactive oxygen by stealing free electrons from the material, and the reaction mechanism is illustrated as follows:

O_2_(g) → O_2_(ads) (7)

O_2_^−^(ads) + e^−^ → 2O^−^(ads) (8)

During this process, the electron concentration decreases, the resistance increases, and a thick electron depletion layer is formed on the surface (Figure 9b).

The oxygen molecules adsorbed on the surface of CDs@ZnO undergo redox reactions at high temperatures, generating free radicals (O^2−^, O^−^, and O_2_^−^). O^2−^, O^−^, and O_2_^−^ dominate at <150 °C, 150–400 °C, and >400 °C, respectively [21]. So, when EG was poured into the test chamber, the O^−^ on the surface of CDs@ZnO reacted fast with EG gas, as seen below:

(CH_2_OH)_2_ + 2O^−^(ads) → H_2_O + CO_2_ + 2e^−^ (9)

In this process, electrons return to the conduction band, the depletion layer of electrons becomes thinner, and the resistance decreases (Figure 9c).

## 4. Conclusions

In this study, a simple one-step hydrothermal process was utilized to manufacture sea urchin-shaped three-dimensional ZnO, which was then compounded with various concentrations of CDs, and the CDs@ZnO sensing material was successfully synthesized and applied to detect EG gas. The gas sensing performance test results show that CDs@ZnO-1 has the best gas sensing performance. Its optimal working temperature is 220 °C, at which the response value to 100 ppm EG is 1356.89. The response of the CDs@ZnO-1 gas sensor to EG does not change significantly after five consecutive cycles of cyclic testing. With the synergistic effect of the heterogeneous structure and abundant oxygen vacancies, the CDs@ZnO gas sensor has high sensitivity, good stability, and reproducibility, which shows its great practical application and provides guiding ideas for the development of high response semiconductor metal oxide sensors.

## Figures and Tables

**Table 1:** Comparison of gas sensitive properties of different materials to EG gas.

Sample	Response (Ra/Rg)	Concentration (ppm)	OOT (°C)	t_a_/t_d_	Ref.
ZnO/rGO Nanoflake	277	100	220	38 s/26 s	[17]
NiO foam@Sn-doped In_2_O_3_ nanowire	160.72	100	240	40 s/55 s	[31]
Zn-doped SnO_2_	90	100	240	66 s/97 s	[32]
NiO-ZnO	142	100	140	31 s/49 s	[26]
CuO-NiO nanotubes	10.4	100	110	15 s/45 s	[10]
**CDs@ZnO-1**	**1356.89**	**100**	**220**	**45 s/9 s**	**This work**

**Table 2:** Linear fits for ZnO and CDs@ZnO samples lnG-(1/T) relationship.

Sample	Formula	Adj. R2	eVs (eV)
ZnO	lnG = −6967.13 × (1/T) − 7.36	0.9824	0.60
CDs@ZnO-1	lnG = −8803.83 × (1/T) − 4.88	0.9853	0.75
CDs@ZnO-2	lnG = −7404.97 × (1/T) − 6.87	0.9791	0.64

## Data Availability

Data are contained within the article.

## Supplementary Materials

The following supporting information can be downloaded at: https://www.mdpi.com/article/10.3390/s25030905/s1, Figure S1. The XRD patterns of ZnO, CDs@ZnO and CDs at 10–30°; Figure S2. (a) The TEM images of CDs@ZnO-1, (b) the Mott-Schottky plots of CDs@ZnO-1; Figure S3. (a) The full spectra of ZnO and CDs@ZnO, (b) the C1s spectra of ZnO, (c) the C1s spectra of CDs@ZnO-1; Figure S4. The Mott-Schottky plots of (a) CDs and (b) ZnO; Table S1. Fitted results of the response curve of ZnO and CDs@ZnO-based sensors for different concentrations of EG gas.
