# Supplementary material for: Ultra-Sensitive Gas Sensor Based on CDs@ZnO
Source: Sensors (Basel). 2025 Feb 2;25(3):905. doi: 10.3390/s25030905 (PMC11820362; doi:10.3390/s25030905)
Supplement: Supplementary file 1 [file sensors-25-00905-s001.zip › sensors-3394154-supplementary.pdf]

Ultra-Sensitive Gas Sensor Based on CDs@ZnO

Shuo Xiao, Zheng Jiao * and Xuechun Yang

School of Environmental and Chemical Engineering, Shanghai University, Shanghai 200444, China
* Correspondence: zjiao@shu.edu.cn

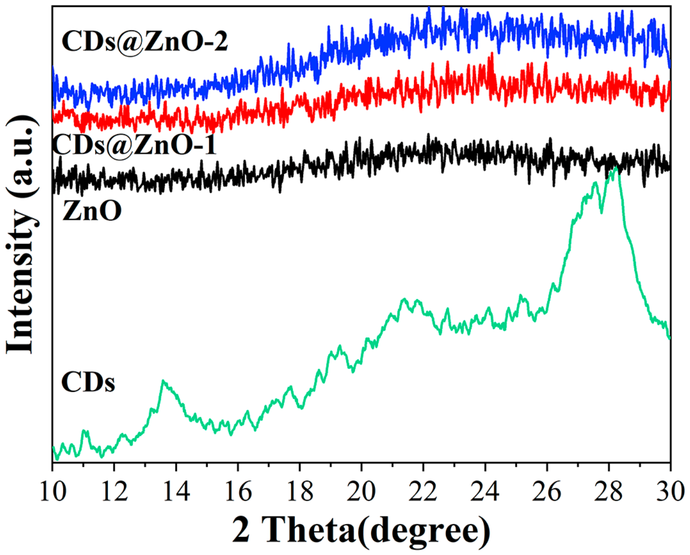

Figure S1. The XRD patterns of ZnO, CDs@ZnO and CDs at 10°-30°.

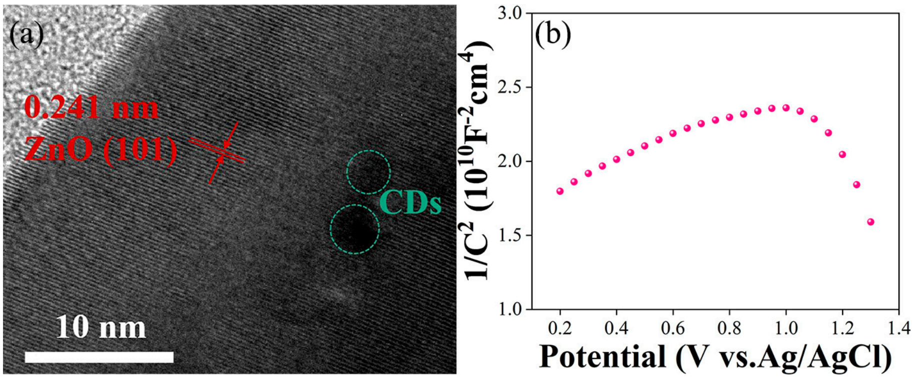

Figure S2. (a) The TEM images of CDs@ZnO-1, (b) the Mott-Schottky plots of CDs@ZnO-1.

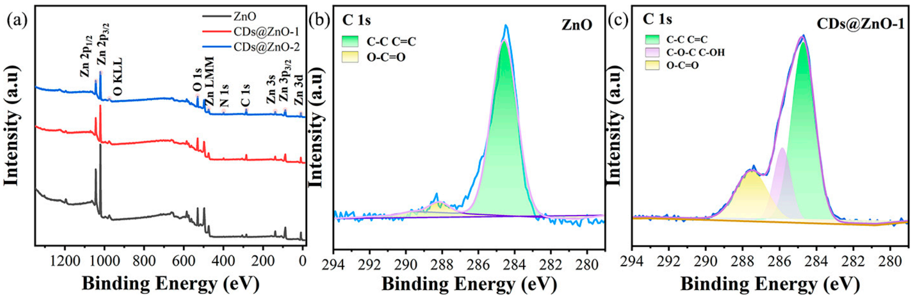

Figure S3. (a) The full spectra of ZnO and CDs@ZnO, (b) the C1s spectra of ZnO, (c) the C1s spectra of CDs@ZnO-1.

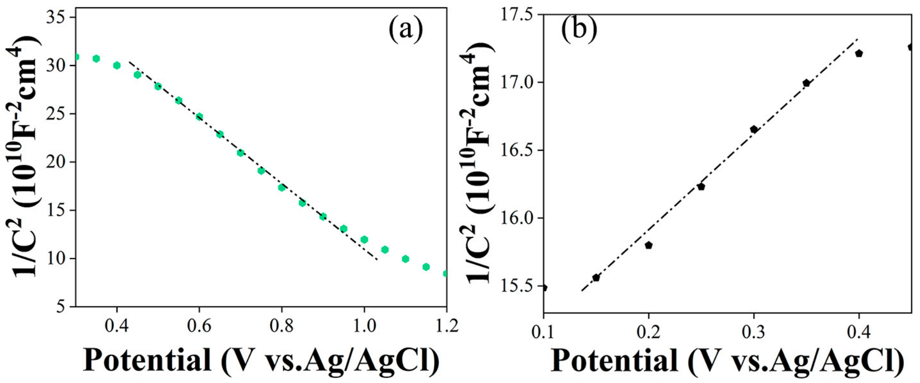

Figure S4. The Mott-Schottky plots of (a) CDs and (b) ZnO.

Table S1. Fitted results of the response curve of ZnO and CDs@ZnO-based sensors for different concentrations of EG gas.

**Sample**	**Fitting results**			
	low concentration	R^2	high concentration	R^2
ZnO	Y = 1.14 + 0.33x	0.99	Y = -0.93 + 0.68X	0.98
CDs@ZnO-1	Y = -14.14 + 8.42x	0.94	Y = -240.28 + 15.73X	0.99
CDs@ZnO-2	Y = -12.06 + 6.72x	0.97	Y = -8.94 + 6.28X	0.99
